# Scenario-based operational evaluation of Median U-Turn intersections across traffic demand levels

**DOI:** 10.1371/journal.pone.0355385

**Published:** 2026-08-12

**Authors:** Abdulrahman Badwes, Saif Alarifi

**Affiliations:** Department of Civil Engineering, King Saud University, Riyadh, Saudi Arabia; Southwest Jiaotong University, CHINA

## Abstract

This paper presents a microscopic simulation modeling of 30 signalized intersections in Riyadh, using detailed datasets on direction traffic volume, lane configurations, and current signal control structure. Six distinct Median U-Turn scenarios were developed, categorized by longitudinal offset from the intersection center ranging from 120–300 meters. These configurations were closely evaluated against the conventional signalized intersection for operational viability. Simulations were executed within PTV VISSIM for both the baseline signalized control and the six MUT alternatives, resulting in more than 3,000 simulation runs to ensure statistical soundness in extracting travel time, delay, and queue length metrics. The final results indicate that the MUT design outperforms the conventional signalized intersection at total entering volumes (TEV) of 4,000 vehicles per hour (vph) or less. Within this range, the results identify optimal offsets of 150m for moderate volumes, 180–210m for high directional splits, and 250–300m for high-intensity demands (>1,200 vph). While MUT designs excel below 4,000 vph, the conventional four-leg signalized intersections consistently emerge as the superior design once this total volume threshold is exceeded.

## Introduction

Rapid urbanization and population growth in Riyadh have increased travel demand and exacerbated congestion across the city’s arterial network. These challenges highlight the need for innovative intersection designs that enhance traffic flow and capacity. As the Median U-Turn (MUT) has demonstrated significant potential, this study evaluates its operational effectiveness under real-world traffic conditions in Riyadh

The road network of Riyadh City in Saudi Arabia comprises more than 4,000 intersections, of which approximately 640 are four leg signalized intersections, according to statistics issued by the [1]. In recent years, the MUT design has been implemented at a limited number of intersections. Most intersections are typically converted into conventional signalized four-leg junctions as traffic increases, often without considering the MUT as a viable alternative.

Previous studies have shown that the MUT design generally results in lower delays; however, traffic volume remains the key determinant of its effectiveness. The functional MUT intersection removes all left-turn conflicts from both the main and secondary roads at the central junction by rerouting vehicles to perform a U-turn before completing their left turn as mentioned at the [2].

This highlights the importance of examining the actual traffic volumes at four-leg signalized intersections. Accordingly, this study investigates 30 signalized intersections and evaluates their conversion into the MUT design at varying distances from the center of the intersection (120, 150, 180, 210, 250, and 300 meters). The objective is to determine, first, whether the current signalized configuration is the most suitable solution, and second, if not, to identify the optimal median opening distance for implementing the MUT design to achieve overall performance improvements.

While various prominent unconventional intersection designs exist in traffic engineering literature such as the Displaced Left-Turn (DLT) and the Diverging Diamond Interchange (DDI) this study specifically focuses on MUT configuration. The selection of MUT design over other alternatives is driven by strict geometric and economic feasibility considerations Implementations of DLTs or DDIs typically demand extensive additional spatial rights-of-way, radical restructuring of signal control infrastructure, and high capital investments. Conversely, MUT design utilizes existing wide urban medians, making it highly compatible with the current geometry of major arterials in Riyadh. This allows for the effective elimination of conflicting direct left-turn phases and maximizes network capacity without requiring secondary land acquisition or complex signal synchronization.

**Fig 1:**
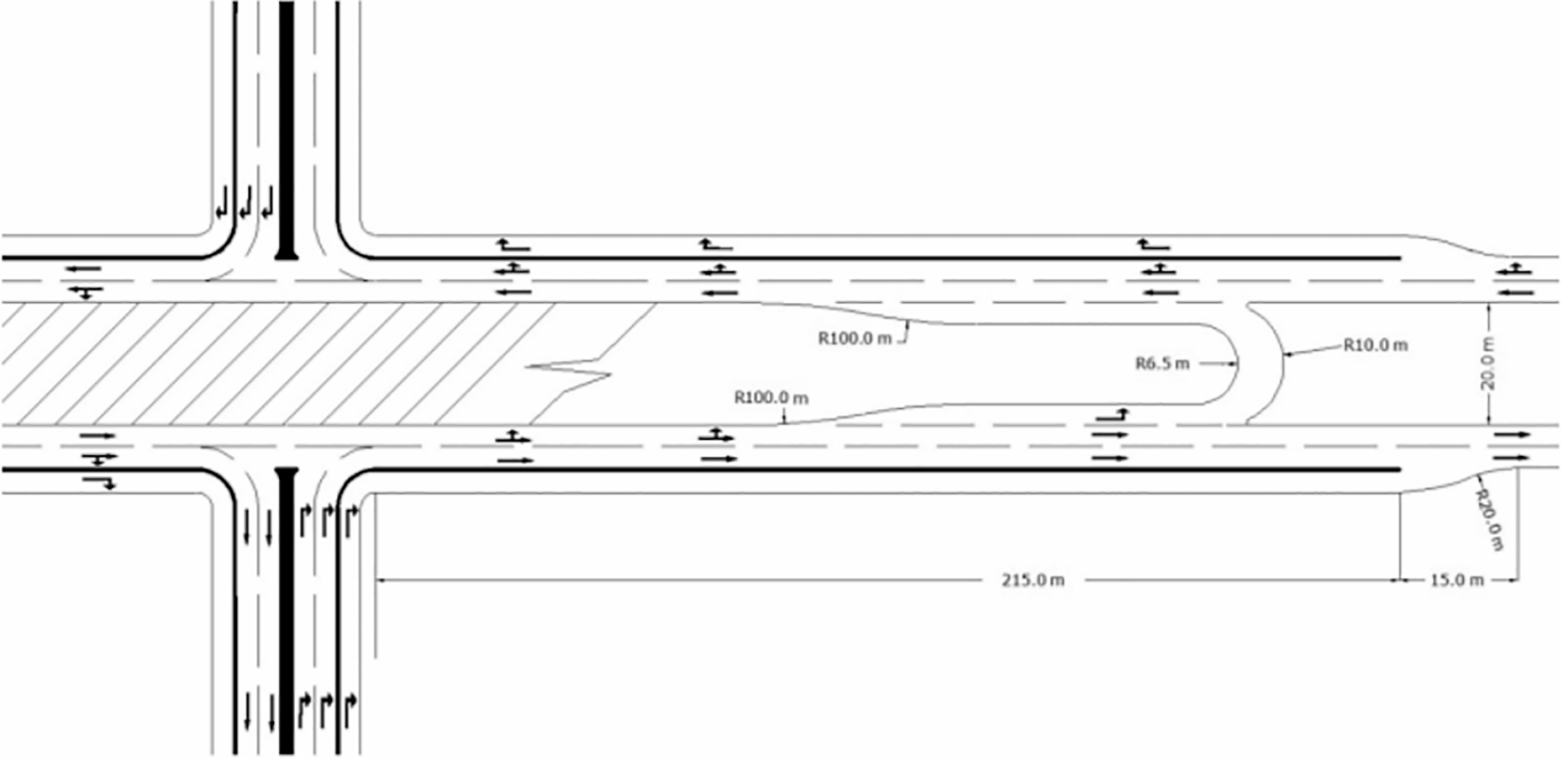
Geometric designs of the unconventional MUT intersections (not to scale).

Due to the noticeable increase in the number of cars according to statistics, it is necessary to have a solution that contributes to reducing congestion, especially at four-leg intersections governed by traffic signals. One of the solutions that has been applied, but unfortunately in a very limited manner, is the Median U-turn as shown in Fig 1 Therefore, it is important to focus on studying and feasibility of the effectiveness of this design within KSA.

The median U-turn (MUT) intersection design has been proposed as an unconventional treatment to alleviate congestion at signalized intersections with heavy left-turn movements. The MUT design was successfully implemented several years ago in many US cities, especially in Michigan, and therefore, is sometimes referred to as the Michigan U-turn design [3].

The findings are intended to support direct selection of the most appropriate design for similar intersections with comparable traffic volumes and main-road lane configurations, enabling the adoption of the MUT concept in both newly planned intersections and in upgrading existing signalized intersections that require enhanced operational efficiency. At-grade intersections represent most intersections within the current road network. However, they have been found to be insufficiently efficient, as evidenced by the growing number of traffic signals, longer signal cycle times, increased queue formation, and higher vehicle delays at these intersections [4].

[5] presented detailed engineering evaluations of the MUT design as an alternative treatment for left turns at signalized intersections., also it mentioned in Saudi highway code 301(SHC 301) that MUT design can reduce the crashes by 20–50% of conventional intersections as shown in [2].

**Fig 2:**
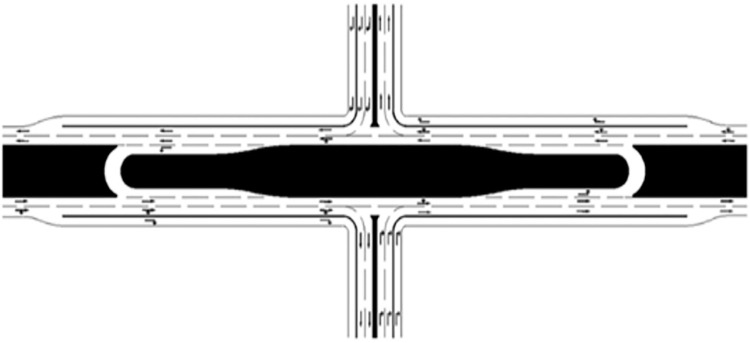
Schematic of typical Unconventional MUT intersection.

Recently, research studies have examined MUT design in terms of safety and types of accidents, proving that this design performs better than conventional intersections. MUT intersections are effective in reducing total, property damage only (PDO), rear-end, and opposite direction sideswipe crashess [6], also [7] provided a design for unconventional U-turn treatments as shown in Fig 2.

Following pioneering federal initiatives, [8] formally established the Median U-Turn (MUT) as a standardized alternative intersection design, noting its historical success in major corridors, where it is traditionally referred to as the Michigan Left [3]. Their guidance outlined essential features such as one-way median crossovers, recommended median widths of about 40–70 ft, and procedures for relocating left-turn movements from major and minor streets. The report highlighted that eliminating direct left turns and the corresponding signal phases improves both operational efficiency and safety by reducing delay and minimizing conflict points.

**Fig 3:**
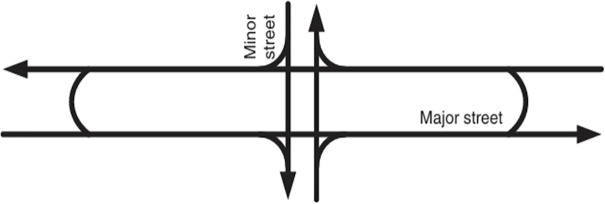
Vehicular movements at typical MUTI.

According to [9], optimizing MUT intersections through lane-based traffic allocation can significantly enhance operational performance. The analysis indicates that relocating left-turns to downstream crossovers and optimizing lane assignments based on approach volumes substantially reduces main-intersection delay, demonstrating the efficiency gains of properly configured MUT designs. In parallel with operational benefits, researchers have investigated the safety performance of MUT intersections and generally found substantial crash reduction relative to conventional configuration. [6] conducted a comprehensive safety evaluation of median U-turn crossover-based intersections using crash data and Empirical Bayes techniques as presented in Fig 3.

Furthermore, [10] evaluated the safety performance of MUT intersections using historical crash data and safety performance functions, finding that MUT designs reduce severe crash frequencies by about 41% and provide superior fatal and injury crash performance compared with conventional signalized and unsignalized intersections. Additionally, [11] investigated the safety implications of MUT intersections in Srinagar by estimating dilemma zone boundaries and spatial critical gaps for U-turning vehicles using cumulative distribution and binary logit methods.

On the operational modeling front, [12] evaluated the new “Shifting Movements” (SM) intersection by developing calibrated microscopic traffic simulation models in PTV VISSIM, proving its superiority in throughput under moderate to heavy minor-road traffic. [13] investigated the feasibility and operational performance of MUT designs in a Middle Eastern context, confirming that converting heavily loaded conventional intersections to MUT configurations significantly improves operational efficiency and reduces fuel consumption and emissions. More recently, [14] evaluated the operational performance of median U-turn intersections using a set of four-leg intersections, where the MUT layout achieved higher capacity thresholds reaching 1,650 veh/h per approach. Reflecting the recent prevalence of VISSIM in simulation research, [15] evaluated hybrid design Continuous Flow Intersection (CFI) and MUT combinations by executing over 1,000 Synchro-optimized scenarios.

Furthermore, modern traffic flow theory emphasizes that vehicular trajectories inside complex intersection conflict zones and weaving corridors display continuous, two-dimensional (2D) behavioral movements governed by human tactical interactions and constrained optimal control [16,17]. In this study, replicating these non-lane-based tactical maneuvers and lateral weaving pressures within VISSIM’s framework, extensive calibration of conflict areas and lateral driving behaviors was rigorously executed across all 30 models.

The geometric placement and physical offset distance of median U-turn crossovers relative to the main intersection represents a critical design threshold that directly dictates the spatial layout required for vehicular lane-changing, acceleration, and deceleration. Recent studies in traffic engineering literature have examined these spatial variations, typically testing rigid, standardized increments such as 100 m, 200 m, and 300 m. However, a major limitation of these recent works is their reliance on hypothetical, synthetic simulation scenarios operating under idealized, symmetrical traffic splits.

The distinct novelty and empirical value of this study lie in overcoming this theoretical limitation through a comprehensive, data-driven network framework. This research evaluates the operational performance of entirely unsignalized, continuous free-flowing MUT configurations across an extensive, highly heterogeneous network of 30 actual urban intersections in Riyadh. By deploying strictly empirical, disaggregated lane by lane and approaching specific peak hour volumes, this study captures real-world stochastic driving behaviors and asymmetric flow pressures. Crucially, the objective of this investigation extends beyond a basic binary comparison between the MUT and conventional four-leg signalized layouts. The ultimate contribution is to synthesize these empirical network responses to identify and isolate the definitive optimal physical offset distance. Consequently, this study provides highway agencies and urban planners with localized, scientifically validated, and directly implementable geometric guidelines for immediate real-world deployment, filling a major practical gap in unconventional intersection implementation frameworks.

## Materials and methods

### Data collection and preparation

**Fig 4:**
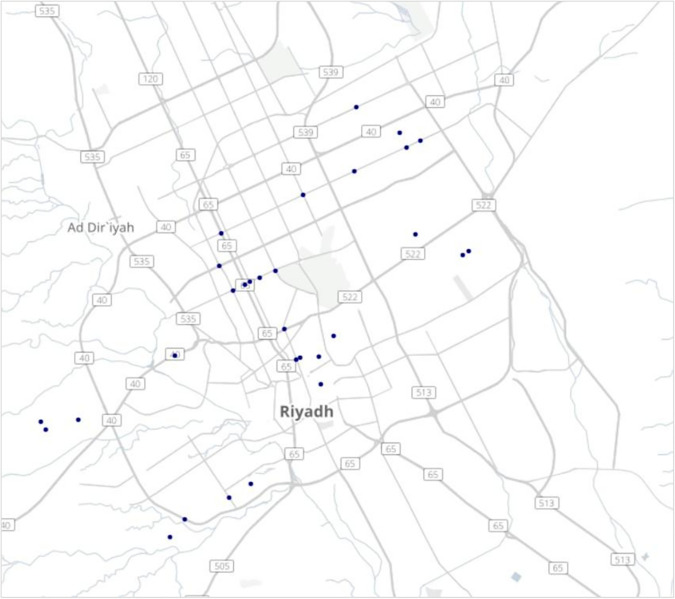
Spatial distribution of the selected intersections within the Riyadh city road network (Source: OpenStreetMap under CC BY 4.0 license).

The first step of the study was to identify a random set of four-legged signalized intersections to ensure a representative sample. A total of 30 intersections across 14 neighborhoods in Riyadh were selected for analysis as illustrated in Fig 4.

Traffic data for these intersections were collected over a full week from Saturday to Friday to capture both weekday and weekend conditions.

Three main types of data were obtained for each intersection:

1. Geometric data: Data regarding the geometrics of the intersection, which are the number of lanes in each approach, width of each lane.
2. Timing report: Details of signal timing such as cycle length, number of phases, Sequences and time of day schedule.
3. Traffic volume: Volume per Lane in each approach calculated for every 15 minutes for a whole week’s duration.

**Fig 5:**
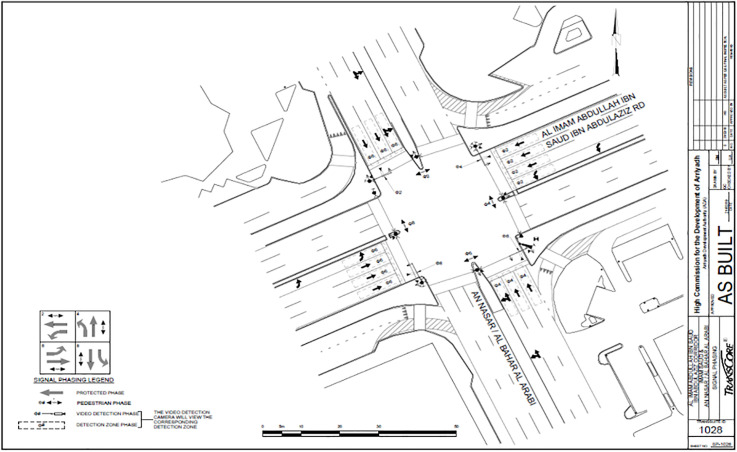
As built design of a sample signalized intersection.

The geometric layout reflects the as-built configuration obtained from the available design documentation and field observations. Fig 5 illustrates the overall geometry of a representative signalized intersection used in the study.

As shown in Table 1, the total cycle lengths for the conventional signalized intersections vary between 100 sec and 120 sec, which were optimized based on the 85th percentile design volumes. Each timing plan was systematically matched with its corresponding time-of-day traffic scenario to represent an optimized baseline network. Crucially, the proposed MUT configurations are entirely unsignalized and free-flowing, operating strictly on localized gap-acceptance parameters. To maintain full operational transparency without cluttering the text, the complete, detailed network timing schedules are provided as a separate document in S3 Table.

**Table 1:** Signal Timing Details of Signalized Intersections.

Pattern Description	Split Table	Phase Yellow (Sec)	Sequences	P2 (sec)	P4 (sec)	P6 (sec)	P8 (sec)
AM Peak WD	1	5.5	2,6,4,8	35	18	25	18
AM Off Peak WD	2	5.5	2,6,4,8	30	15	22	15
MD Peak WD	3	5.5	2,6,4,8	32	18	25	18

*WD, Weekday;MD, Midday*

**Fig 6:**
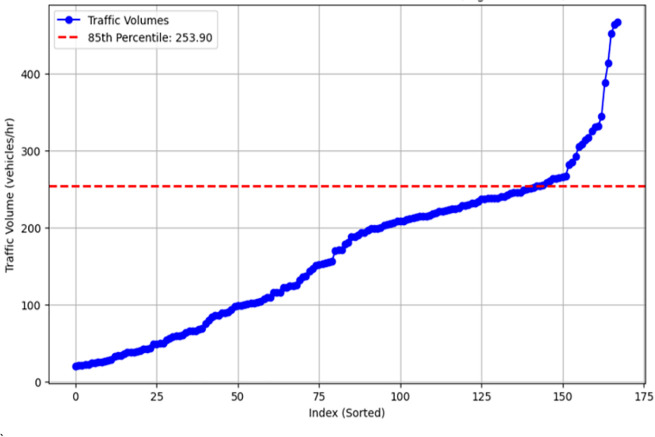
Distribution of hourly traffic volumes with indicated 85th percentile.

Traffic volume data for each lane of the subject approaches were obtained from the Royal Commission for Riyadh City (RCRC). The dataset comprised continuous recordings taken at 15-minute intervals over a one-week period. For analytical purposes, these data were aggregated into hourly flow rates. To account for temporal fluctuations and establish a robust standard for analysis, the 85th percentile was adopted as the design volume (DV). The cumulative distribution of these hourly traffic volumes, highlighting the exact positioning of the selected 85th percentile threshold, is visually demonstrated in Fig 6.

This ensures that the designs are evaluated under near-peak conditions while excluding extreme outliers, consistent with established transportation engineering practices, such as [18]. The following Data in Table 2 represents the vehicle class distribution in Riyadh City [19], based on the latest RCRC statistics. These parameters were incorporated to ensure the simulation accurately reflects real-world conditions and current operational environments.

**Table 2:** Distribution of Vehicle Classes.

Vehicle Class	Percentage
Private Cars	77.2%
Private Buses/Vans	16.1%
Taxis/Limousines	3.7%
Public Buses	2.2%
Heavy Trucks	0.8%

The evaluation of operational boundary conditions of the proposed MUT configurations, the critical role of left-turn volume proportions across the 30 studied intersections was thoroughly analyzed. As detailed in the comprehensive statistical breakdown provided in S1 Table, the left-turn demand varied significantly across the network, ranging from minor balanced distributions to heavy, asymmetric flows exceeding 35% of the total approach volume.

To ensure a comprehensive operational assessment of the proposed MUT configuration, the simulation scenarios were designed based on the critical 85th percentile design volume extracted from the master field dataset. This master dataset inherently includes continuous traffic counts spanning both weekdays and weekends across the studied network. Utilizing the 85th percentile threshold provides a mathematically conservative control layout that ensures geometric resiliency under peak congestion periods, where unconventional intersection configurations are most warranted. Furthermore, while the simulation framework isolates this design peak, a wide multi-range spectrum of traffic demand levels (encompassing light, medium, and heavy ranges) is naturally evaluated within the study. This is driven by the vast demographic and functional diversity of the 30 intersections studied which are provided in S1 Table. Consequently, evaluating this highly heterogeneous 30-intersection network effectively captures the operational sensitivity of the unsignalized MUT layout across all critical traffic density ranges, omitting the need for redundant lower-demand off-peak or weekend sub-scenarios that consistently operate under under-saturated, low-delay conditions.

**Fig 7:**
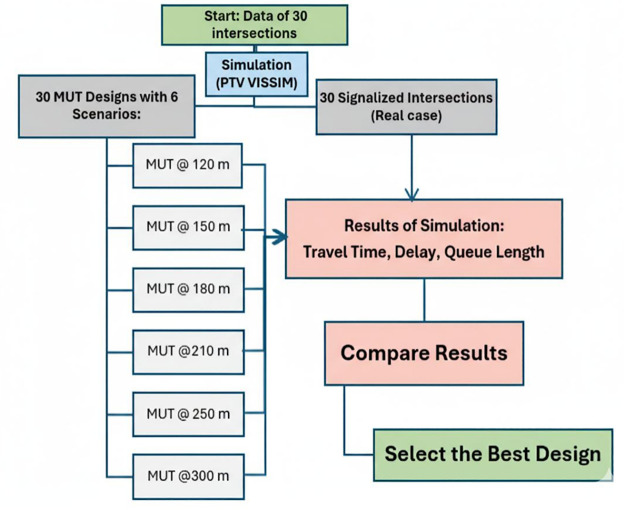
Methodology for the study.

The methodology adopted in this study is divided into two primary stages. The first stage involves obtaining traffic data from the competent authority, while the second stage consists of conducting traffic simulation to derive the performance outcomes. The overall research workflow, beginning from data acquisition and proceeding through the simulation process until the generation of results as presented in Fig 7.

The process begins by collecting traffic data for 30 real-world four leg signalized intersections located in Riyadh, Kingdom of Saudi Arabia. These intersections are modeled using realistic traffic simulation PTV VISSIM to assess key performance indicators, including travel time, delay, and queue length. Subsequently, six MUT design scenarios were developed with offsets of 120 m, 150 m, 180 m, 210 m, 250 m, and 300 m as detailed in Table 3. Each scenario was simulated to extract performance metrics-specifically travel time, delay, and queue length- which were then compared against the baseline (Signalized intersection). This comparison aims to identify the most efficient MUT design configuration or determine the operational superiority of the conventional signalized design.

**Table 3:** Six Scenarios of MUT Intersection Design.

Scenario	MUT Distance (m)	Reference	Justification
S1	120	AASHTO Green Book	FHWA lower recommended bound
S2	150	AASHTO/ FHWA	FHWA & AASHTO optimal for moderate
S3	180	FHWA (AIIR)	FHWA upper bound for general conditions
S4	210	MDOT (GEO-670)	Extend spacing to test travel time
S5	250	NCDOT	For high-speed Roads
S6	300	FHWA (RCUT)	Test for long rerouting impact

*AASHTO, American Association of State Highway and Transportation Officials; FHWA, Federal Highway Administration;AIIR, Alternative Intersection Information Report; NCDOT, North Carolina Department of Transportation.*

### Vissim model development

**Fig 8:**
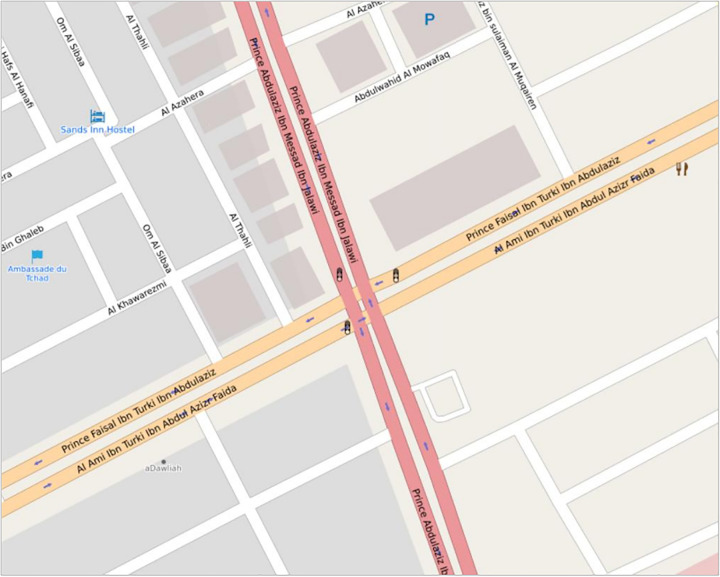
View of real signalized intersection (Source: OpenStreetMap under CC BY 4.0 license).

The microsimulation model was constructed in PTV VISSIM to replicate the existing signalized intersection; Fig 8 illustrates a representative intersection used in this study.

**Fig 9:**
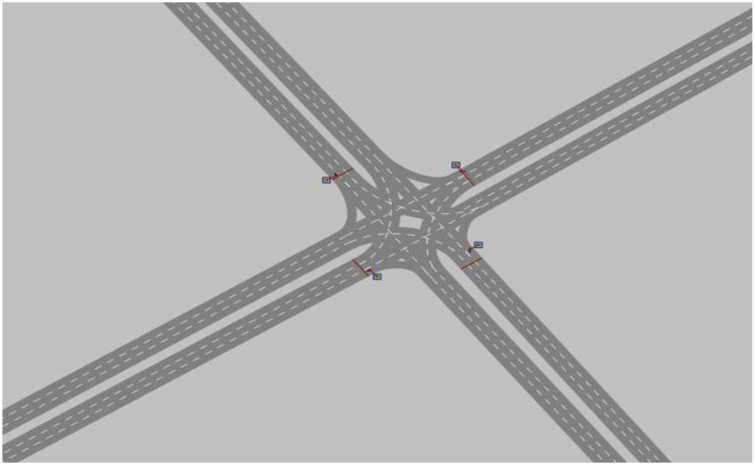
VISSIM simulation of a sample real case signalized intersection.

It is a four-legged signalized intersection from which traffic data were collected, applied, and then converted into a simulation model to replicate real-world conditions and produce the corresponding results. The model was based on actual geometric characteristics, including lane configuration and approach widths, as shown on Fig 9.

**Fig 10:**
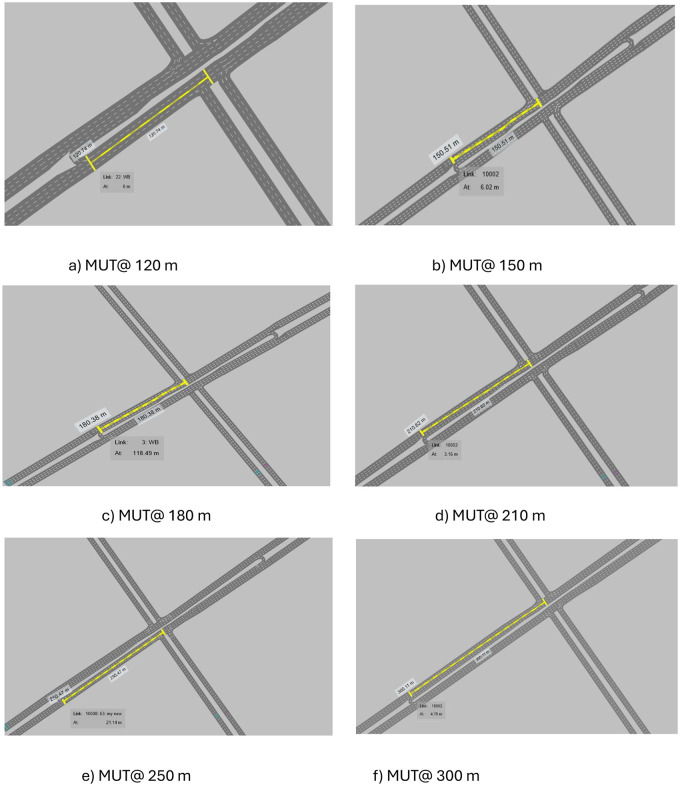
VISSIM simulation of six scenarios of unconventional MUT intersection design.

To evaluate operational improvements, six alternative design scenarios were developed by varying the MUT offset distance (120, 150, 180, 210, 250, and 300 m), as illustrated in Fig 10.

The underlying operational mechanism across the 120 m to 300 m offset distances is dictated by weaving dynamics and spatial segregation. Shorter offsets (120–180 m) compress the zone available for U-turning vehicles to execute lane changes, creating severe weaving disturbances and forcing sharp driving angles that disrupt the major continuous stream, resulting in localized queue formation and high delays. Conversely, optimal larger offsets (210–300 m) provide a sufficient spatial buffer that acts as an extended weaving zone, allowing U-turning vehicles to decelerate smoothly into the dedicated storage space without exerting returning-flow pressure on the main corridor.

To ensure structural and operational feasibility across the evaluated corridors, the geometric design of the proposed MUT crossovers was strictly aligned with international standards and local field constraints. The inner and outer turning radius at the crossover points were developed in accordance with the (AASHTO) criteria, ensuring safe clearances for standard design vehicles. The baseline median widths across the 30 studied intersections vary dynamically from 3.64 m to 10.0 m, mirroring real-world constraints in Riyadh’s urban network. At locations characterized by narrow medians where the physical space was insufficient to safely accommodate the tracking paths of executing vehicles, Loons (localized external pavement widenings) were strategically deployed. These configurations eliminate geometric conflicts and support continuous, non-staged maneuvers. Furthermore, to maximize directional capacity and mitigate potential queue build-ups, each MUT crossover was structured with dual turning lanes (2 lanes), with each individual lane possessing a standardized width of 3.5 m. This configuration facilitates efficient gap acceptance and accommodates heavy peak-hour traffic volumes.

By maintaining identical lane geometry and traffic demand across all scenarios, observed changes in performance metrics could be attributed solely to the geometric modifications.

Unlike conventional median U-turn designs that rely on secondary traffic signals to regulate crossover movements, the proposed MUT configurations evaluated in this study are completely unsignalized and free-flowing. Traffic movements at the crossovers are governed purely by geometric features and continuous flow based on natural gap acceptance (modeled via calibrated conflict areas and priority rules in PTV VISSIM), thereby completely eliminating signal cycle overhead and phase-based delays.

To ensure the empirical validity of the operational analysis, the simulation inputs in PTV VISSIM were structured to mirror the exact traffic characteristics collected during field surveys. The traffic demand distribution across the 30 studied intersections is strictly unbalanced, exhibiting highly asymmetric flows between major arterials and intersecting minor legs, as well as directional peak splits. The disaggregated hourly volumes capturing all four directional movements (Northbound, Southbound, Eastbound, and Westbound) are comprehensively detailed in S1 Table to support applicability assessments. The traffic demand utilized represents the 85th percentile design hour volume. The temporal boundary conditions for the simulation network consist of a 2-minute warm-up period to populate the network links and establish a realistic steady-state traffic profile, followed by 1 full hour of peak simulation runtime for data collection. To minimize stochastic simulation noise and ensure statistical confidence, each geometric scenario was executed across 5 independent replication runs utilizing distinct random seeds, with the arithmetic mean of the outputs extracted to analyze queue formation, vehicular delay, and corridor travel times.

### Model calibration and validation

The VISSIM micro-simulation model was rigorously calibrated and validated to ensure it accurately replicates the stochastic traffic conditions of the study area in Riyadh. Initially, the vehicle composition and class distribution were tuned using official empirical data and statistics provided by the Royal Commission for Riyadh City (RCRC).

To enhance the realism of the simulation, specific attention was given to driving behavior parameters. The lane-changing distance was adjusted to 200 m, providing vehicles with sufficient spatial opportunity for optimal lane selection, which is critical for the operational performance of complex intersection designs. To account for the stochastic nature of microsimulation and ensure stability, each scenario was run for 15 simulation replications with different random seeds, minimizing random variability and guaranteeing statistically significant outputs.

The model’s validity was quantitatively assessed by comparing simulated throughput volumes against observed field data. The network-wide validation results demonstrated an exceptionally high degree of modeling accuracy, with more than 95% of all individual turning movements across the 30 intersections achieving a GEH score below the critical threshold of 5.0, as summarized in Table 4. The GEH statistics were utilized as the primary performance indicator to measure the “goodness-of-fit” between the model and reality, calculated as follows:

**Table 4:** Summary of model validation Results.

Validation Metric	Achieved Value	Target Benchmark
Average GEH Score	1.84	< 5.0
Percentage of Movements (GEH < 5.0)	95.2%	> 85%


$$\begin{array}{c} \;GEH=\sqrt{\frac{\text{2}(M-C{)}^{\text{2}}}{M+C}} \end{array}$$ (1)

Where M represents the simulated hourly volume and C represents the observed field volume. According to international benchmarks, a model is considered validated if the GEH value is less than 5.0 for at least 85% of the movements. Critically, to guarantee that secondary performance measures such as vehicular delay, queue length formation, and corridor travel times accurately reflect field realities under complex, unsignalized weaving conditions, advanced driving behavior calibration was performed. The Wiedemann 74 car-following and lane-changing parameters were systematically customized to match the dense urban driving characteristics of Riyadh, in alignment with the Saudi Highway Code 301 (SHC 301). Specifically, the average standstill distance (ax) was calibrated to 1.2m, and the safety distance factors (bx_add_ and bx_mult_) were adjusted to 2.0 and 3.0, respectively, forcing the simulation engine to generate realistic, behaviorally underpinned delays and queue formations across all baseline models.

## Results and discussion

In this study, a total of 30 signalized intersections were analyzed using microscopic traffic simulation to evaluate operational performance indicators, including travel time, vehicle delay, and queue length. Each signalized intersection, representing the existing condition, was systematically converted into multiple alternative design scenarios based on the MUT concept. Six MUT offset distances ranging from 120 m to 300 m were examined for each intersection to assess their impact on traffic operations.

### Operational performance

**Fig 11:**
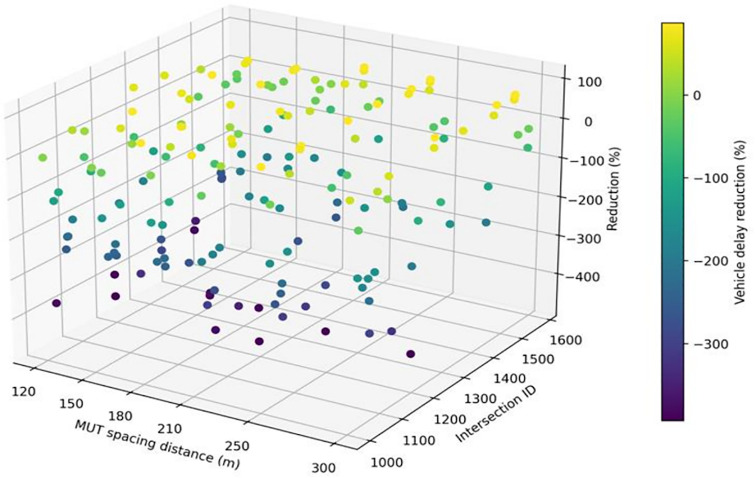
Scenario-based comparison of vehicle delay performance between MUT and signalized.

The scenario-response characteristics of vehicle delay reduction resulting from the application of MUT designs with different spacing distances, assessed in relation to signalized control throughout the network under study, are shown in Fig 11 in order to separate the impact of geometric modification from intersection demand variations.

The observed dispersion of delay reduction values across intersections shows significant spatial heterogeneity in MUT performance, indicating that local traffic patterns and geometric constraints have a significant impact on the operational effectiveness of a given spacing distance. While some spacing scenarios show significant delay reductions for a subset of intersections, other locations show significant performance degradation, as indicated by large negative values.

**Fig 12:**
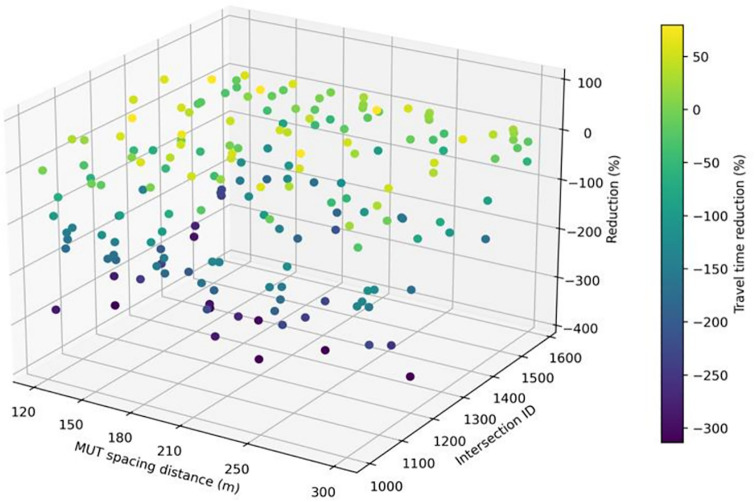
Scenario-based comparison of travel time performance between MUT and signalized intersection designs.

The lack of universally ideal MUT spacing and the danger of applying uniform design without site-specific evaluation are highlighted by this contrast. As a result, the figure supports the need for robust-based and aggregated evaluations to find spacing configurations that offer consistent network-level benefits as opposed to isolated local improvements It demonstrates that delay reduction is not a constant function of spacing; rather, it is highly sensitive to unbalanced approach volumes. The scenario-response behavior of travel time reduction associated with various MUT spacing distances in relation to signalized control across the examined intersections is shown in Fig 12. In order to isolate the impact of the MUT geometric spacing on travel time performance, each point represents a within-intersection comparison between the signalized configuration and a particular MUT scenario.

While negative values show longer travel times under MUT operation, positive values show shorter travel times. The findings show significant variation in travel time response between intersections, suggesting that network connectivity and local traffic demand patterns have a significant impact on MUT efficacy. While some locations experience significant performance degradation, especially at intermediate and longer spacing distances, other locations consistently reduce travel times for a subset of intersections. This dispersion emphasizes the need for site-specific assessment and aggregated performance evaluation when choosing the best MUT design parameters and shows that improvements in travel time are not always guaranteed by increasing MUT spacing. This demonstrates the Travel time is affected by traffic demand and the distance of offset of MUT where yellow point gives the best performance of the MUT distance.

**Fig 13:**
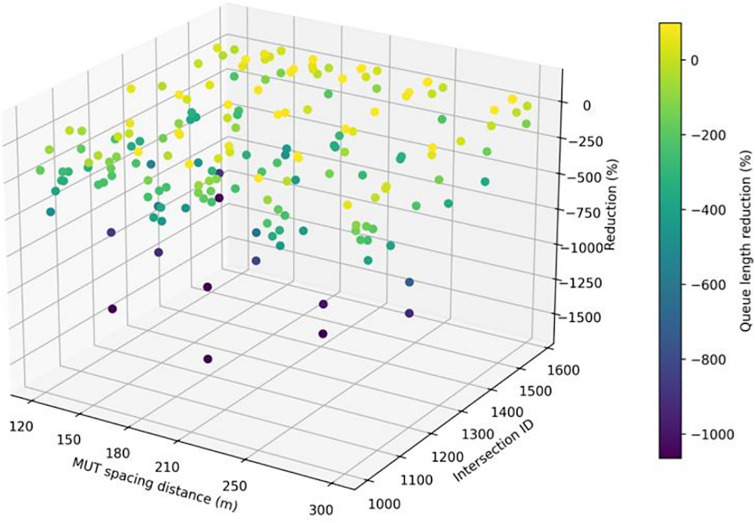
Scenario-based comparison of queue length performance between MUT and signalized intersection designs.

Positive values indicate queue reduction, whereas negative values reflect queue amplification under MUT operation. Fig 13 represents the substantial dispersion in queue response across intersections; with several scenarios producing extreme queue increases for specific locations. This pronounced variability suggests that queue performance under MUT designs is particularly sensitive to local demand imbalance, storage availability, and weaving dynamics introduced by U-turn movements.

It should be noted that some MUT scenarios produced very large negative percentage changes in delay and travel time relative to the conventional signalized intersection. These values primarily occurred when the baseline delay or travel time under the signalized condition was relatively small. In such cases, even moderate absolute increases in delay or travel time under a specific MUT configuration can result in large relative percentage changes. Therefore, the percentage-based results should be interpreted together with the corresponding absolute delay and travel time values provided in Supplementary S2 Table, which offer a more comprehensive assessment of operational performance.

**Fig 14:**
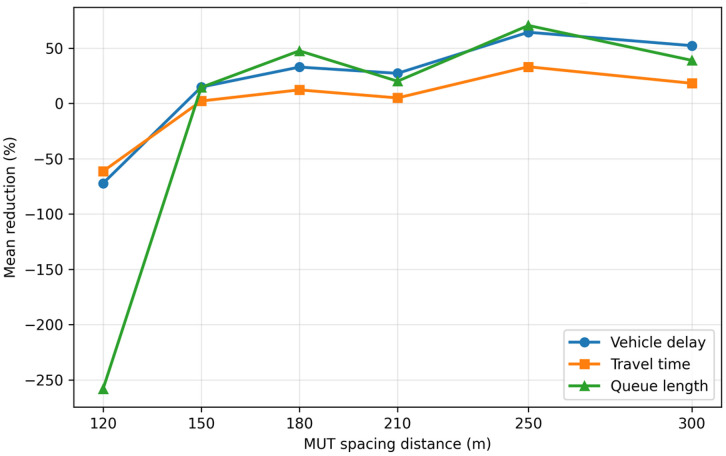
Conditional mean performance (Successful cases only).

While certain spacing distances result in modest queue reductions for some intersections, the presence of severe queue growth in others highlights that queue length is the most vulnerable performance measure among the evaluated indicators. These findings emphasize that MUT spacing selection must carefully account for available storage and traffic distribution, as inappropriate spacing may lead to critical operational failures despite improvements in delay or travel time. Each point represents the performance change of one intersection under a specific MUT scenario relative to its signalized baseline. Results for all 30 intersections are included; however, only selected intersection identifiers are displayed on the axis as shown in Fig 14 which presents a combined comparison of the mean percentage reduction in vehicle delay, travel time, and queue length, calculated only for cases where performance improvement was achieved relative to signalized control where they were 13 out of 30 intersections.

The conditional mean percentage reduction in vehicle delay, travel time, and queue length for MUT spacing distances, Unlike the overall results, this figure highlights the potential benefits of MUT implementation under favorable conditions by excluding unsuccessful outcomes. The results indicate that, when MUT designs are effective, spacing distances between 180 m and 250 m yield the highest average improvements across all three performance measures. However, the contrast between the unconditional and conditional results underscores that these benefits are not universally guaranteed and depend strongly on intersection-specific characteristics.

### Demand threshold analysis for MUT applicability

**Fig 15:**
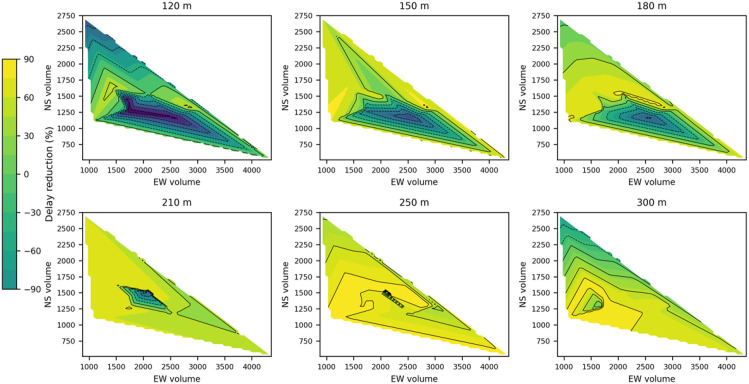
Effect of directional traffic volumes on delay reduction under different MUT spacing distances.

Fig 15 illustrates the relationship between directional traffic volumes and the delay reduction achieved under different Median U-Turn spacing scenarios. Directional demand is represented along the horizontal axes, where the east–west traffic volume (WB + EB) and the north–south traffic volume (NB + SB) jointly define the operating condition of each intersection. The color gradient and contour lines indicate the percentage change in vehicle delay relative to the signalized baseline, while each panel corresponds to a specific MUT spacing distance (120–300 m).

The results demonstrate that the delay performance of MUT designs is strongly dependent on both traffic demand distribution and spacing distance. Shorter spacings (120–150 m) exhibit limited or negative delay reduction under high traffic volumes, particularly when demand is concentrated along one direction, reflecting storage constraints and intensified weaving effects. In contrast, intermediate spacings (180–210 m) provide broader regions of delay reduction, especially under moderate and more balanced directional volumes, indicating a more favorable interaction between U-turn spacing and traffic flow dynamics. Larger spacings (250–300 m) offer localized improvements for certain demand ranges; however, these benefits are not consistently observed across all volume combinations, suggesting diminishing returns when spacing increases beyond a threshold. Overall, the figure confirms that no single MUT spacing distance universally minimizes delay across all traffic conditions. Instead, optimal MUT performance emerges from a combined consideration of spacing distance and directional traffic demand, underscoring the necessity of site-specific and volume-based design evaluation rather than uniform application of MUT configurations.

### Development of the decision support GUI

**Fig 16:**
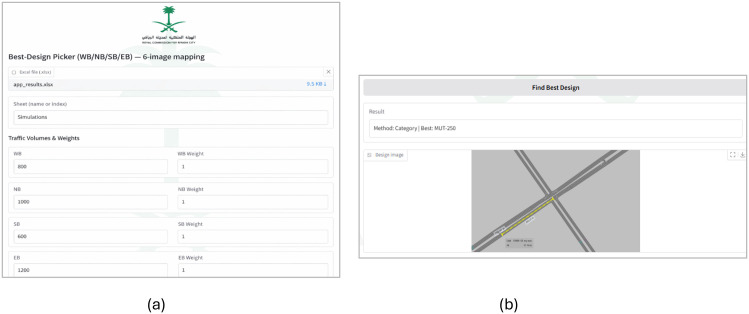
Graphical user interface shows inputs (a) and outputs (b).

To enhance the practical applicability of the proposed approach, a lightweight decision-support interface was developed to demonstrate real-time use of the trained model. The interface allows users to input traffic volume in all approaches as veh/h and immediately obtain the best design for the intersection as shown in Fig 16-part a and b respectively.

The interface was implemented using a web-based framework within a cloud-based Python environment by incorporating a machine learning based neighborhood selection (ML nearest-neighborhood) within the appropriate spatial context. This implementation is provided solely as a proof of concept not part of modeling results.

## Conclusions

This study utilized VISSIM microsimulation to evaluate the operational performance of MUT designs against four-leg signalized intersections. By analyzing crossover offsets from 120 m to 300 m across various Total Entering Volumes and directional splits, the research identifies the optimal geometric configurations required to maximize urban traffic flow under diverse demand scenarios. For total entry volume below 4000 v/h, the Conventional MUT design configurations specifically those with crossovers at 120, 150, 180, 210, 250, and 300 m outperformed the conventional four-leg signalized intersection. MUT at 150 meters was the best when volume in two opposite approaches are moderate (500–900 vph) and others are low (less than 500 vph). MUT at 180 meters was the best when the volume in one directional movement was high (more than 900 vph) and the other was moderate (i.e., WB is high and EB is moderate) and other directional movements are moderate for the two approaches. The 210-meter MUT offset was most effective under two conditions: when volumes were high for both approaches of a single directional movement (e.g., high WB and EB) with low volumes on the perpendicular approaches, or when all four approaches maintained moderate volumes. The 250-meter MUT offset proved most effective when two opposing approaches experienced high volumes (approaching 1,200 vph) while others remained moderate, or when a single approach was high and the remaining three were moderate. At higher intensities, the 300-meter offset was optimal for scenarios where opposing approaches both exceeded 1,200 vph alongside moderate volumes elsewhere. Notably, once the total entering volume (TEV) exceeded 4,000 vph, the conventional four-leg signalized intersection emerged as the superior design.

## Supporting information

S1 Table Comprehensive traffic demand inputs and multi-approach left-turn proportions for the 30 studied intersections. (XLSX)

S2 Table Absolute delay & travel time values. (XLSX)

S3 Table Schedule of the signal timing configurations. (PDF)
