# Supplementary material for: Scenario-based operational evaluation of Median U-Turn intersections across traffic demand levels
Source: PLoS One. 2026 Aug 12;21(8):e0355385. doi: 10.1371/journal.pone.0355385 (PMC13465966; doi:10.1371/journal.pone.0355385)
Supplement: Supplementary file 1 [file pone.0355385.s003.pdf]

**Intersection:**	Al Imam Abdullah Ibn Saud Ibn Adulaziz Rd @ An Nasar				
**Controller ID:**	1028.6	**Channel:**	106	**Drop:**	1
**System:**	TransCore TransSuite TCS				
**Controller Type:**	Peek ATC-1000 3.26				
**TransCore Unified Controller Manager 23.2.6**					

Cycle, Offset, Split (in Seconds)

Pattern #	Pattern Description	Split Table	Cycle Length	Offset	P2	P4	P6	P8
**1	AM Peak WD	1	0	0	35	18	25	18
**2	AM Off Peak WD	2	0	0	30	15	22	15
**3	MD Peak WD	3	0	0	32	18	25	18
**4	PM Peak WD	4	0	0	35	18	25	18
**5	PM Off Peak WD	5	0	0	32	18	28	22
**6	AM Peak WE	6	0	0	22	15	22	15
**7	MD Peak WE	7	0	0	25	15	25	25
**8	PM Peak WE	8	0	0	35	18	25	18
**9	AM Peak WE	9	0	0	22	15	22	15
**10	MD Peak WE	10	0	0	25	15	25	15
**11	PM peak WE	11	0	0	35	18	25	18
**12	Overnight	12	0	0	20	15	20	15
13	IMP-150-CL	13	150	0	*50	25	50	25
14	IMP-180-CL	14	180	0	*60	30	60	30
**15	Special Assist Plan-1	15	0	0	50	25	55	35
**16	Special Assist Plan-2	16	0	0	60	35	70	30
17	APOP Test Plan-1	17	105	0	*30	20	35	20
**20	Light Cycle-1 (PD-P2/WB)	20	0	0	30	15	22	15
**21	Light Cycle-2 (PD-P6/EB)	21	0	0	28	15	24	15
**22	Light Cycle-3 (Bal)	22	0	0	26	15	25	15
**23	Medium Cycle-1 (PD-P2/WB)	23	0	0	35	18	25	20
**24	Medium Cycle-2 (PD-P6/EB)	24	0	0	32	18	28	22
**25	Medium Cycle-3 (Bal)	25	0	0	28	18	28	25
**254	Free	0	0	0	60	60	60	60

* = Coordinated Phase | ** = the values used for the plan are green values and do not include clearances

Sequences

Sequence #	Rings
	1
1	2,6,4,8

Phase Timings

	2	4	6	8
Minimum Green (sec)	10	10	10	10
Passage (Sec)	1.0	1.0	1.0	1.0
Phase Maximum (Sec)	60	60	60	60
Phase Yellow (Sec)	5.5	5.5	5.5	5.5

Phase Timings

Phase Red (Sec)	3.5	3.5	3.5	3.5
Phase Yellow Warning (sec)	0	0	0	0
Ped Walk (Sec)	5	5	5	5
Ped Clear (Sec)	7	7	7	7
Phase Startup	X			

Time Of Day Schedule

Day Plan	Days of the Week	Pattern Description	Action #	Pattern #	Sequence	Begin	End
1	SUN-THU	Overnight	12	12	1	00:00	05:30
1	SUN-THU	AM Peak WD	1	1	1	05:30	09:30
1	SUN-THU	AM Off Peak WD	2	2	1	09:30	11:00
1	SUN-THU	MD Peak WD	3	3	1	11:00	16:00
1	SUN-THU	PM Peak WD	4	4	1	16:00	00:00
2	FRI	Overnight	12	12	1	00:00	10:00
2	FRI	AM Peak WE	6	6	1	10:00	14:00
2	FRI	MD Peak WE	7	7	1	14:00	16:00
2	FRI	PM Peak WE	8	8	1	16:00	00:00
3	SAT	Overnight	12	12	1	00:00	10:00
3	SAT	AM Peak WE	9	9	1	10:00	14:00
3	SAT	MD Peak WE	10	10	1	14:00	16:00
3	SAT	PM peak WE	11	11	1	16:00	00:00
